# Prioritizing risk genes as novel stratification biomarkers for acute monocytic leukemia by integrative analysis

**DOI:** 10.1007/s12672-022-00516-y

**Published:** 2022-06-30

**Authors:** Hang He, Zhiqin Wang, Hanzhi Yu, Guorong Zhang, Yuchen Wen, Zhigang Cai

**Affiliations:** 1 Department of Hematology, Tianjin Medical University General Hospital, Tianjin, China; 2 Department of Rheumatology, Tianjin Medical University General Hospital, Tianjin, China; 3 The Province and Ministry Co-Sponsored Collaborative Innovation Center for Medical Epigenetics, Department of Pharmacology, School of Basic Medical Science, Tianjin Medical University, Tianjin, China

**Keywords:** Biomarker, Acute myeloid leukemia, Transcriptome, *t*-SNE, Stratification and prognosis, Venetoclax

## Abstract

Acute myeloid leukemia (AML) is a blood cancer with high heterogeneity and stratified as M0–M7 subtypes in the French-American-British (FAB) diagnosis system. Improved diagnosis with leverage of key molecular inputs will assist precisive medicine. Through deep-analyzing the transcriptomic data and mutations of AML, we report that a modern clustering algorithm, *t*-distributed Stochastic Neighbor Embedding (*t*-SNE), successfully demarcates M2, M3 and M5 territories while M4 bias to M5 and M0 & M1 bias to M2, consistent with the traditional FAB classification. Combining with mutation profiles, the results show that top recurrent AML mutations were unbiasedly allocated into M2 and M5 territories, indicating the *t*-SNE instructed transcriptomic stratification profoundly outperforms mutation profiling in the FAB system. Further functional data mining prioritizes several myeloid-specific genes as potential regulators of AML progression and treatment by Venetoclax, a BCL2 inhibitor. Among them two encode membrane proteins, *LILRB4* and *LRRC25*, which could be utilized as cell surface biomarkers for monocytic AML or for innovative immuno-therapy candidates in future. In summary, our deep functional data-mining analysis warrants several unappreciated immune signaling-encoding genes as novel diagnostic biomarkers and potential therapeutic targets.

**Supplementary Information:**

The online version contains supplementary material available at 10.1007/s12672-022-00516-y.

## Key points

1. *t*-SNE instructed transcriptomic stratification discriminates FAB subgroups of AML.
2. Potential association of the prioritized genes with Venetoclax resistance at single-cell resolution.

**Supplementary Information:**

The online version contains supplementary material available at 10.1007/s12672-022-00516-y.

## Introduction

Acute leukemia is a group of clonal hematologic malignancies originated from aberrant hematopoietic stem and progenitor cells (HSPCs) with significant heterogeneity. Acute myeloid leukemia (AML) is the most common type of acute leukemia in adults. Based on hematopoietic cellular morphology, immunophenotype, cytogenetic and genetic alterations, AML has been stratified into several subtypes. Although the AML diagnosis has been just recently updated by WHO and ELN, the FAB diagnosis system is still the most popular guideline in clinic since 1970s and classifies AML into 8 subtypes, M0–M7 [1–3].

AML with certain monocytic differentiation (M-AML) accounts for about 30% of all AML cases. It is diagnosed primarily by morphological and immunophenotypic characteristics, such as the degree of bone marrow differentiation and leukemia blast cell count. M-AML mainly includes acute myelomonocytic leukemia (FAB M4) and acute monoblastic leukemia (FAB M5) [4]. Since M-AML is prone to extramedullary infiltration, the accurate diagnosis of M-AML patients at an earlier stage remains a challenge. Lacking sensitive and specific immature monocyte markers is another reason that M-AML is difficult in diagnosis. Monocyte markers in M-AML have been reported, such as CD56, CD64, CD45, CD11b, CD14 and CD117. However, these traditional markers could not label immature monocytes of M-AML [4, 5]. Identifying novel markers of immature and mature monocytes is therefore necessary to improve diagnosis. Increasing numbers of AML study cohorts and transcriptomic/genomic datasets provide invaluable sources for searching such diagnosis and risk stratification biomarkers.

At present FAB M3 subtype patients (acute promyeloid leukemia, APL) could be treated by arsenic trioxide combined with all-trans retinoic acid dual induction therapy to achieve clinical cure. However, treatment of other FAB subtype patients remains challenging [1–3]. In addition to standard chemotherapy and hematopoietic stem cell transplantation, new treatments are emerging, such as immunotherapy, cellular therapy, and targeted therapy, but with a considerable number of patients do not achieve complete remission after treatment or eventually relapse. Of note, M-AML patients (FAB M4 and M5) were more likely to develop bone marrow and extramedullary recurrence after stem cell transplantation than other subtypes (FAB M0, M1 and M2) [6]. Furthermore, recent studies report that the resistance to Venetoclax, a BCL2 inhibitor, is significantly higher in FAB M5 than that in other FAB subtypes. Considering that Venetoclax has been recently approved and starts to be widely used in the treatment of refractory AML, decoding the novel regulators and mechanisms of the drug resistance is also demanded [7–9].

To overcome the heterogeneity of AML in diagnosis and improve the therapeutic efficiency of Venetoclax, identifying novel molecular biomarkers of FAB_M5 therefore appears urgent and important. The present study integrates clinical information, mutation profiles, AML patient transcriptome of pooled samples or single cell samples, datasets of Venetoclax drug tailoring and knowledge from animal AML models. We finally prioritized several novel biomarkers for AML patients, especially for FAB M5 subtypes. Their prognosis values were also verified at the pooled-cell or single-cell resolution.

## Methods

### Source of transcriptomic, genetic and clinical datasets

All of the datasets in the study had been published and are available for download online. See Supplemental Table 1 for the DOI address of the discovery datasets. If not stated elsewhere, gene expression level was recorded and normalized as Reads Per Kilobase of exon model per Million mapped reads (RPKM) in RNA-seq datasets for comparing analysis. Initial discoveries were focused on the full datasets of The Cancer Genome Atlas Leukemia (TCGA_LAML) and Beat_AML [10, 11], including mutation profiles, clinical information, survival status, transcriptome (bulk RNA-seq) and Venetoclax drug sensitivity (area under the curve, AUC). As outlined in Figure S1, five cohorts of AML patients from different clinical centers and three murine models for AML diseases were included for prioritizing risk genes and pathways: TCGA_LAML, Beat_AML, KI_AML, Leucegene_AML and UHN_AML. To validate the expression profile of risk genes in healthy and leukemia samples, RNA-seq and ATAC-seq datasets were collected in the platforms of both pooled samples and single-cell samples. See also Supplemental Table 1 for the DOI address of validation datasets.

### Clustering and visualizing transcriptomic data using PCA, *t*-SNE and UMAP algorithms in R

All of the visualization results were generated in R (version 4.0) using proper packages. See Supplemental Table 2 for the software information of R packages for clustering and following analysis. Since that Microarray or RNA-seq based transcriptomic data are high-dimension matrix, three popular dimension reduction algorithms were implemented to visualize the data in the 2-dimentional (2D) setting, one is the classical Principal Component Analysis (PCA) and the other two are developed and applied wildly in singe-cell RNA-seq (scRNA-seq) data analysis: *t*-distributed Stochastic Neighbor Embedding, *t*-SNE; Uniform Manifold Approximation and Projection, UMAP. Although PCA visualization in 2D failed to stratify M2, M3 and M5, both *t*-SNE and UMAP have good performance in dimension reduction and visualization. For convenience, only *t*-SNE displays in 2D were reported in the study for visualizing transcriptome at the single patient level. Once the 2D matrix of *t*-SNE plotting is determined, other information (i.e. mutation profiles or gene expression levels) were concatenated to the matrix and further projected to the same *t*-SNE plot by R package ggplot2.

### Prioritizing differentially expressed genes (DEGs) and calculating their hazard ratio (HR)

DEGs in the comparison between *NPM1*_Group1 vs. *NPM1*_Group2 or in the comparison between FAB_M5 vs. FAB_M2 were calculated by the R package limma. Top 50 variable genes, volcano plots, Venn diagram and correlation analysis of DEGs were performed in R using proper packages. See Supplemental Table 2 for the software information of R packages. To calculate DEGs’ HR value, clinical information including patient survival status and following-up days was downloaded from TCGA. Overall survival (OS) was used as a prognostic endpoint. In survival analysis using the TCGA dataset, patients with following-up days less than 30 days were excluded. The R packages survival and survminer were used for univariate Cox hazard regression analysis to evaluate OS-related risk genes. Gene expressional level-based Kaplan–Meier survival plots and the p values were also calculated and visualized accordingly.

### GSEA and KEGG analysis for prioritizing essential biological pathways

The R package clusterProfiler and the software GSEA 4.1.0 were used for enrichment analysis of altered biological pathways. Supplemental Table 2 for the software information and original publications. Alterations with normalized enrichment score (NES) > 1.5 or < −1.5 and p < 0.05 and were considered statistically significant.

### Validation of prioritized genes in healthy and AML samples at the pooled or single cell level

Expression of the prioritized genes in monocytic cells or progenitors were validated using public datasets of both healthy and leukemic samples, including in the platforms of single cell or pooled RNA-seq and ATAC-seq (Assay for Transposase-Accessible Chromatin with high throughput sequencing). See the Supplemental Table 1 for the DOI address of these datasets. Correlation of the genes in AML risk stratification was also validated using a longitudinal scRNA-seq dataset of a patient with two stages: Diagnosis (Dx) and Relapse (Rl). The risk value of each cell in the patient was inferred by R package Scissor. After normalization of the Dx/Rl dataset by R package harmony, identities of each cluster were annotated by R package celldex. Plots of gene and protein expression level at single cell level were generated by R package Seurat4.

### Human cell lines

Human normal 293 T cells and AML cell lines THP‑AML (THP‑1) were purchased from The Type Culture Collection of the Chinese Academy of Medical Sciences (Shanghai, China). AML cells were maintained in RPMI‑1640 medium (Thermo Fisher Scientific) containing 10% Fetal Bovine Serum (Thermo Fisher Scientific). 293 T cells were maintained in DMEM supplemented with 10% FBS. We maintained cell lines at 37˚C in a 5% CO2 cell culture incubator and tested all cell lines routinely for mycoplasma contamination.

### Construction of the lentiviral and cell transfection.

Plasmids expressing human *LILRB4* were cloned into the pSin-Flag (Addgene) vector (GenScript). Positive clones were screened using ampicillin as a selection pressure (Sigma‑Aldrich) and identified by Sanger sequencing on recombinant plasmid. To produce lentiviral particles, 4 × 10^6^ 293 T cells in a 10-cm dish were co-transfected with LILRB4-pSin-Flag, psPAX2 and pVSV-G (Addgene) plasmids. The supernatant containing viral particles was harvested twice at 48 h and 72 h after transfection, then filtered through Millex-GP Filter Unit (0.45 μm, Millipore). Viral particles were concentrated in 4 °C high speed centrifuge at 12,000 rpm for 6 h, resuspended in DMEM and stored at −80 °C until use.

A total of 2.5 × 10^5^ cells/well were plated in a final volume of 1 ml culture media. For infection, THP-1 cells were plated onto 6-well plates at 2 × 10^5^ cells/well and infected with lentiviral stocks at a multiplicity of infection of 100 in the presence of polybrene (8 µg/ml; Sigma-Aldrich). Puromycin at a final concentration of 1 µg/ml (Sigma‑Aldrich) was added to the media 72 h after transfection to select for stably transfected cells.

### Real-time qPCR

Total RNA was extracted from AML cell lines using TRIzol^®^ (GenStar). Reverse transcription was performed at 37 °C for 15 min and then 85 °C for 15 min using a StarScript III RT Kit.

(GenStar). The relative *LILRB4* expression levels were calculated and normalized using the comparative quantification cycle 2^−ΔΔCt^ method relative to an endogenous control *ACTB*. SYBR qPCR Mix (GenStar) was used to detect and quantify the expression of the target gene. The following primers were used: *ACTB*, 5′-CTGGAACGGTGAAGGTGACA-3′ (forward) and5′-CGGCCACATTGTGAACTTTG-3′ (reverse); *LILRB4*, 5′- CATCCATGACAGAGGACTATGC-3′ (forward) and 5′- GGGCTGAAAGGGTGGGTTTA-3′ (reverse).

### Cell growth analysis.

The cells were counted, and the cell suspension was diluted to a final concentration of 2 × 10^4^/ml. The cell suspension was seeded into a 96-well plate (100 µl/well). Subsequent to adding 10 µl Cell Counting Kit‑8 (CCK‑8; Dojindo) solution to each well, the plates were incubated for 1 h in the incubator and the absorbance at 450 nm was measured with a microplate reader after0, 24, 48, 72 and 96 h. Cell proliferation and survival curves were plotted based on the absorbance values.

### Drug treatment

THP-1 cells were incubated for appropriate time in RPMI 1640- medium supplemented with 10% FBS and titrated concentrations of Venetoclax (Selleck Chemicals). Venetoclax is 10 mmol/L in DMSO. To calculate the IC-50 of the drug, drug treatments with different concentrations were designed. The treatments in Fig. 7D were repeated at least 3 times.

### Statistical computations

All statistical calculations in the study were performed with proper R packages. Sizes of samples and p values were labeled accordingly. If not stated elsewhere, difference with p < 0.05 was recognized as statistically significant.

### Code availability

All scripts for computational analysis and visualization were written in R and available upon reasonable request. The study did not generate new software or algorithms.

## Results

### Stratification of AML patients by t-SNE clustering transcriptomic features

**Fig. 1:**
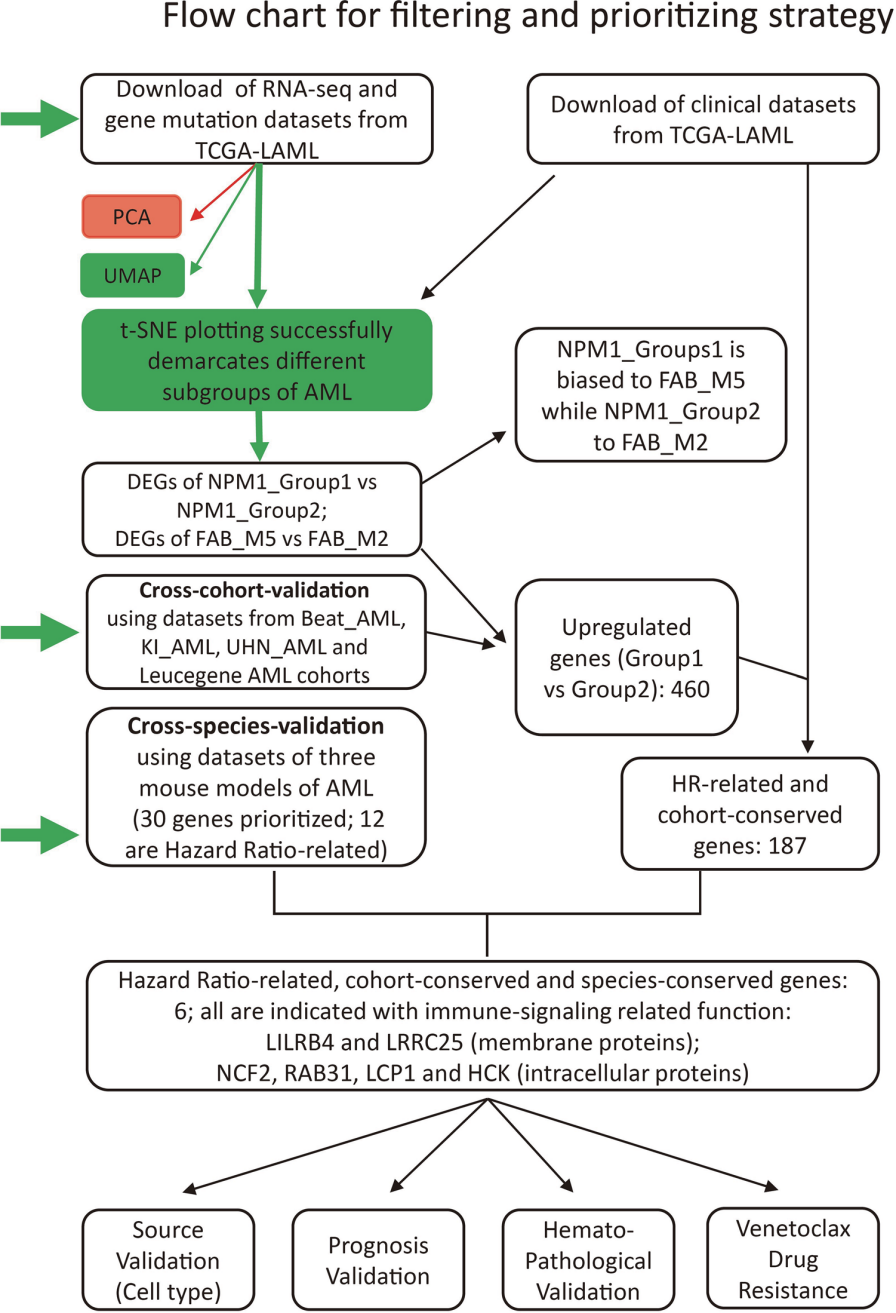
Overview of the working flow of the study. See Methods and main text for details. All datasets are publicly available and from three major sources (horizontal green arrows): datasets of human AML patients from (1) TCGA_LAML, and (2) from other centers such as BeatAML, Europe (KI) and Toronto Hospital (UHN), and (3) datasets of murine models for AML diseases. To setup the working flow for a deep functional data-mining systematically, bulk RNA-seq and clinical datasets of TCGA-LAML were initially downloaded and analyzed for clustering along with FAB subtypes and gene mutation profiles. In consistent to Merstung et al., 2015, transcriptomic data-based clustering the AML patients by PCA algorithm failed to stratify well the FAB subtypes in a 2D setting. However, we found that t-SNE and UMAP algorithms outperform PCA and could at least demarcate well three major categories of AML: (1) Primitive, FAB_M0/M1/M2; (2) Promyeloblast, FAB_M3; and (3) Monocytic, FAB_M4/M5 (Fig. 2A and data not shown). Due to that only few samples of FAB_M6 and FAB_M7 are available in TCGA_LAML, these two rare subtypes were not included in the study. Secondly, the genomic mutation profiles of the top 3 AML-related genes, NPM1, DNMT3A and FLT3 (FLT3-ITD and FLT3-KTD), were also projected to the t-SNE plots but failed to stratify FAB_M2 and FAB_M5 (see also Fig. 2B). NPM1-positive patients were therefore divided into NPM1_Group1 and NPM1_Group2 for following analysis. Thirdly, correlation analysis between NPM1_Group1 and FAB_M5 and searching key features (genes and pathways) of these patients was carried on as shown in the chart and in the main text. The main outcomes of the analysis were arranged and shown in Figs. 2, 3, 4, 5, 6 After several rounds of validation and prioritization, we finally identified six genes that likely play important roles for diseases progression in NPM1_Group1 and/or FAB_M5 patients: LILRB4, LRRC25, NCF2, RAB31, LCP1 and LCK. Interestingly all encode immune signaling components and two of them encodes membrane proteins: LILRB4 and LRRC25

Datasets of AML generated in TCGA and other centers such as BeatAML and Leucegene provides invaluable resources with easy access for further comprehensive data-mining. In previous studies, we and other laboratories have reported numerous *TET2*- and *PTPN11*-related murine models of myeloid leukemia [12–15]. However, the mechanisms of the disease onset and progression are still largely unknown. It is also unknown to what extent those animal models recapture the features of human leukemia. We hypothesize that the *TET2*- and *PTPN11*-related animals manifest features of FAB_M4/M5 rather than FAB_M0/M1/M2 since they are also hot mutations in chronic myelomonocytic leukemia and juvenile myelomonocytic leukemia (CMML and JMML). To extract key features of FAB_M4/M5, we performed the present study in silico as outlined in Fig. 1. Our analysis integrated various aspects of clinical information and basic research. The results were also validated comprehensively as briefed in Fig. 1.

**Fig. 2:**
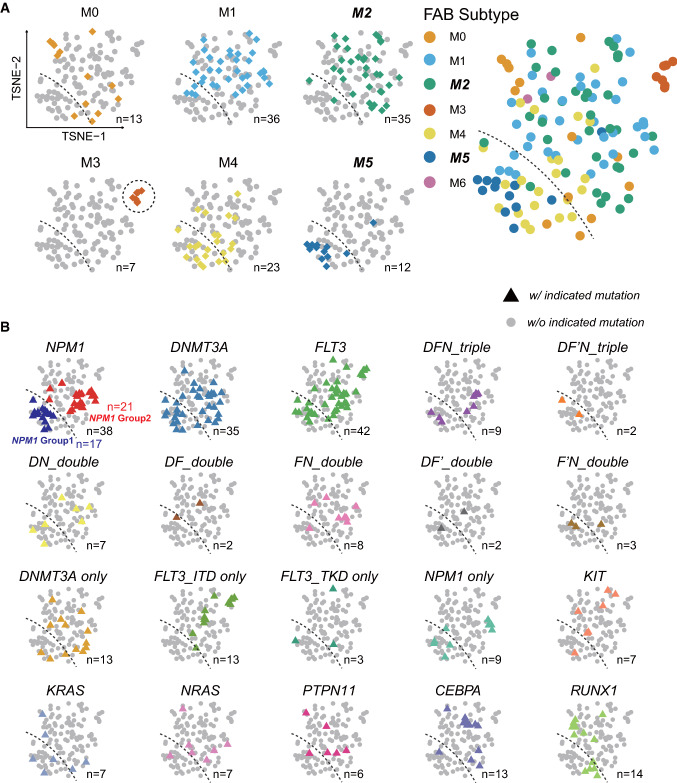
*t*-SNE plotting of high-dimensional transcriptomic functional data successfully demarcates the territory of AML FAB-M2, M3 and M5 patients. **A** In the t-SNE plots of TCGA-LAML RNA-seq datasets, patients diagnosed as FAB-M2, M3 and M5 subtypes were allocated with distinct visible boundaries. Each dot represents a single AML patient. Each color of the dots represents an FAB subtype of AML (M0–M5). The dashed line marks a visible boundary between M5 and M2. **B** AML patients with mutations in *NPM1, DNMT3A and FLT3* were unbiasedly allocated in the M2 and M5 subtypes. Mutation profiles including the top three mutated genes in AML [*NPM1、DNMT3A、FLT3* (*FLT3*^*ITD*^* and FLT3*^*KTD*^)] and their combinations were projected to the t-SNE plots (colored triangle shapes; other patients without indicated mutations are indicated as gray dots for background). *DFN_triple*, combination of mutations in *DNMT3A, FLT3*^*ITD*^ and *NPM1; DF’N_triple*, combination in *DNMT3A, FLT3*^*KTD*^ and *NPM1*; DN, combination in *DNMT3A* and *NPM1; DF,* combination in *DNMT3A* and *FLT3*^*ITD*^*; FN,* combination in *FLT3*^*ITD*^ and *NPM1; DF’, combination in DNMT3A and FLT3*^*KTD*^*; F’N,* combination in *FLT3*^*KTD*^ and *NPM1.* Note that the dichotomous distribution was observed in AML patients with mutations in *NPM1*, *DNMT3A* and *FLT3*, among many other mutation profiles. For convenience in following studies, patients with mutations in *NPM1* were designed as *NPM1*_Group1 and *NPM1*_Group2 according to the dashed line. The total number of AML patients in the plots is n = 126. The numbers of subgroups are shown in each panel of **A** and **B** respectively

As an initial discovery, transcriptomic datasets in the form of RNA-seq (gene expression level normalized as RPKM) of AML patients were downloaded from TCGA [11]. Instructed by the study of Gerstung et al.[16], we first analyzed the AML transcriptomic data by the PCA dimension reduction algorithm to test if any clusters can match FAB subtypes. Using dimensional embedding scores of only two components (PC1 and PC2 for 2D plotting), we found that PCA plots did not classify well FAB subtypes (data not shown). We then performed the dimension reduction by *t*-SNE and UMAP, which are now widely implemented in single cell RNA-seq analysis (scRNA-seq) [17]. Surprisingly, we found that the two modern dimensional reduction algorithms for clustering successfully demarcates M3, and also M2 and M5 (Fig. 2A). The FAB M3 subtype of AML is also known as the APL and has unique histological and molecular features in clinic. Interestingly, the patients in M3 subtype are clustered well at the top-right corner in the *t*-SNE display (Fig. 2A, dashed circle, brick-red diamonds, n = 7 for M3 patients; each diamond represents a single patient). Furthermore, the “territory” of M2 and M5 turns almost distinct as separated by the dashed line in the plot (n = 35 for M2 and n = 12 for M5). Of note, two M2 patients were allocated bellowed the dashed line and one M2 patient above the dashed line as three outliers (3 out of 47 in total). When considering other subtypes, we found that the territory of M4 is biased to that of M5 while the territory of M0/M1 biased to M2 (Fig. 2A). Due to few cases of M6 and M7 available in the TCGA datasets, they were not included in the present study. The outstanding intuitive performance of *t*-SNE in clustering AML patients was also validated using other datasets from Microarray platforms or from other centers or cohorts (Figure S1A, C and D). These results suggest that transcriptomic inputs-based *t*-SNE clustering successfully demarcates M3, M2 and M5 subtypes in AML stratification.

To test if any AML recurrent mutations bias to certain *t*-SNE clusters or FAB subtypes, we projected the mutation information to the same *t*-SNE plot. Top recurrent AML mutations in genes which involves in epigenetic regulations, cell signaling, transcriptional programming were selected for such analysis: *NPM1, DNMT3A, FLT3, KIT, KRAS, NRAS, PTPN11, CEBPA* and *RUNX1*. When combination of mutation was not considered, patients with mutations in *KIT*, *NRAS*, *PTPN11* and *CEBPA* appear to be allocated in the region above the dashed line (territory of M2) (Fig. 2B; n = 7, 7, 6, 13 respectively). However, the mutations in the top 3 AML-related genes *NPM1, DNMT3A* and *FLT3* were randomly allocated in the two sides of the dashed line (Fig. 2B; n = 38, 35, 42 respectively). Most of their combinations are also unbiasedly allocated to the two regions (Fig. 2B). Since the patient number with these three mutations is greater than n = 30, the dichotomy in distribution is persuasive. Indeed, we also validated that the *NPM1*-positive AML patients were stratified into two clusters in the BeatAML cohort (Figure S1B). For convenience in following studies, the AML patients with *NPM1* mutations were divided into two subgroups in the TCGA dataset, namely *NPM1*_Group1 whose territory bias to FAB_M5, and *NPM1*_Group2 whose territory bias to FAB_M2 (Fig. 2B; n = 17, 21 for *NPM1*_Group1 and Group2 respectively).

### Correlation between NPM1_Group1 and FAB-M5 AML patients at the levels of gene and biological pathway

**Fig. 3:**
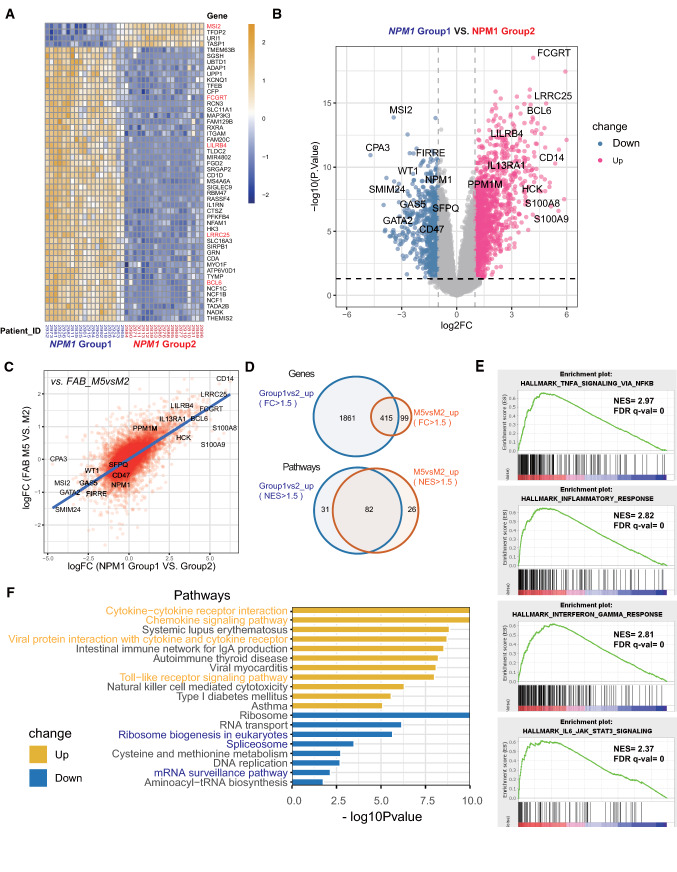
Overlaps of differentially expressed genes (DEGs) and altered pathways between *NPM1* _Group1 and FAB-M5 patients. **A** Heatmap of top 50 variable genes between *NPM1*_Group1 and *NPM1*_Group2 (n = 17 and 21 respectively). The short names of Patient IDs are shown in the below. **B** Volcano plot of DEGs with significant changes between *NPM1*_Group1 and *NPM1*_Group2 (fold change > 2 or < −2 with p < 0.05). **C** Correlation plot of gene expression changes of two transcriptomes from *NPM1*_Group1 vs. Group2 comparison and from FAB_M5 vs. M2 comparison. Each red dot represents a gene. X-axis, logFC value (log2 value of fold change) from *NPM1*_Group1 vs Group2 comparison; Y-axis, logFC value from the comparisons of FAB_M5 vs. M2. The blue line is a linear regression fitting based on all genes’ logFC value. **D** Venn diagrams of overlapped DEGs and pathways in FAB-M5 and *NPM1*_Group1 patients. **E** Representative up-regulated pathways in FAB_M5 compared to FAB_M2 patients assessed by Gene Set Enrichment Analysis (GSEA). **F** Representative up-regulated and down-regulated pathways in FAB_M5 compared to FAB_M2 patients assessed by Kyoto Encyclopedia of Genes and Genomes (KEGG) analysis. Yellow bars, up-regulated pathways; blue bars, down-regulated pathways

To analyze the potential correlation and extract essential features between *NPM1*_Group1 and FAB-M5 in biological aspects, we compared the molecular differences between *NPM1*_Group1 vs. Group2 and also between FAB_M5 vs. M2 at the level of genes and biological pathways. Using *NPM1*_Group2 AML patients as the control group, differentially expressed genes (DEGs) were identified and as visualized in the heatmap (for top 50 variable genes) and volcano plot (for fold change value and p value) (Fig. 3A and B). Among the top DEGs, genes known for HSPC maintenance were upregulated in *NPM1*_Group2 and highlighted, such as MSI2 and GATA2 (Fig. 3A and B). Accordingly, genes known for mature monocytes were upregulated in *NPM1*_Group1 and highlighted, such as S100A8, S100A9, CD14, BCL6 and LILRB4.

The correlation of *NPM1*_Group1 vs. Group2 and FAB_M5 vs. M2 was then analyzed at the entire transcriptome level (N = 17,848 genes included). As shown in Fig. 3C, upregulated DEGs in *NPM1*_Group1 vs. Group2 turns to be among the upregulated DEGs in FAB_M5 vs. M2. More than 400 upregulated DEGs and more than 80 upregulated biological pathways in *NPM1*_Group1 (and FAB_M5) were extracted (fold change or enrichment score > 2 or < -2 and p < 0.05), including TNF signaling pathway, inflammatory pathway, interferon gamma pathway and IL6/STAT pathway. In contrast, pathways involved in ribosome-related transcriptional regulation and RNA surveillance were enriched in *NPM1*_Group2 (and FAB_M2) (Fig. 3D, E and F). These results suggest that *NPM1* Group1 and Group2 of AML patients indeed have distinct transcription features, and that *NPM1* Group1 patients are correlated to FAB M5 subtypes and *NPM1* Group2 patients are correlated to FAB M2 subtype.

### Cross-cohort validation of dichotomy of NPM1 positive AML patients

**Fig. 4:**
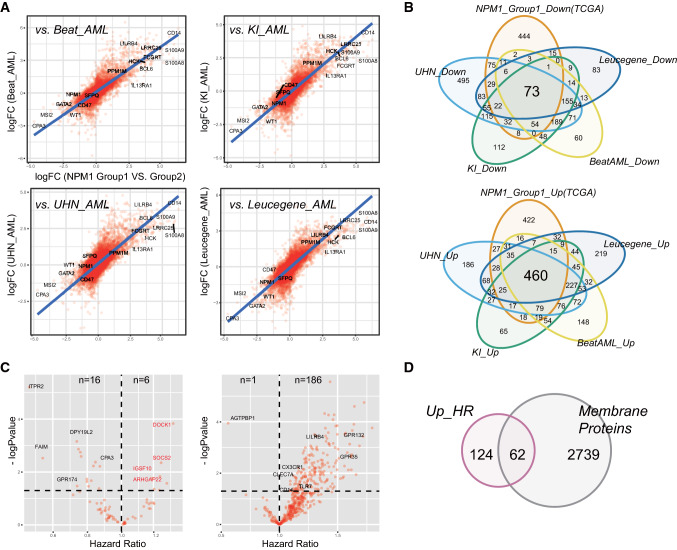
Cross-cohort validation of dichotomy of *NPM1*^+^ AML patients and prioritization of essential genes in NPM1_Group1 by risk correlation and prognostic analysis. **A** Correlation plots of gene expression changes of transcriptomes from *NPM1*_Group1 vs. Group2 comparison of TCGA_LAML datasets and from other sources of AML datasets: Beat_AML, KI_AML, UHN_AML and Leucegene_AML. Each red dot represents a gene. X-axis, logFC value from NPM1_Group1 vs Group2 comparison in the TCGA_LAML dataset; Y-axis, logFC value from the comparisons in other four datasets respectively. The blue line is a linear regression fitting based on all gene’s logFC value. **B** Venn diagrams of up-regulated genes and down-regulated genes in the five AML datasets. Only DEGs with fold change > 2 or < -2 and p < 0.05 were included. **C** Volcano plots of co-downregulated and co-upregulated genes after Hazard Ratio (HR) analysis between gene expression level and survival status. Among 73 co-downregulated genes, 6 are positively correlated to AML patient survival while 16 are negatively correlated. Among 460 co-upregulated genes, 186 are positively correlated to AML patient survival while only 1 is negatively correlated. **D** A third of HR-related co-upregulated genes encode membrane proteins

Rather than clustering by *t*-SNE and UMAP, based on a probabilistic expression deconvolution method, it has been recently reported that the PERT algorithm also stratify *NPM1*-positive AML patients into two groups: the primitive group and the committed group [18]. To verify the dichotomy of *NPM1* positive patients, we conducted similar correlation analysis as above between *NPM1*_Group1 vs. Group2 and FAB_M2 vs. M5. As shown in Fig. 4A, the DEGs extracted from *NPM1*_Group1 vs. Group2 are well correlated with that from Beat_AML, KI_AML, UHN_AML and Leucegene_AML datasets. Furthermore, as show in Fig. 4B, 73 downregulated and 460 upregulated DEGs were extracted by overlapping analysis (Fold change > 2 or < −2, p < 0.05). These DEGS were further prioritized by hazard ratio analysis using TCGA-LAML dataset (Fig. 4C). Out of 73 downregulated DEGs, the HR value of 6 genes is greater than 1, including *SOCS2* and *DOCK1*, suggesting their expression levels are positively correlated with survival status (Fig. 4C). Out of 460 upregulated DEGs, the HR value of 86 genes including *CLEC7A*, *LILRB4*, CX3CR1 and GPR132 is greater than 1 and only 1 is smaller than 1 (Fig. 4C). Interestingly, one third of HR-related upregulated DEGs (n = 62) encodes membrane proteins, provide fertile resource for protein labeling and stratification by flow cytometry in the future (Fig. 4D).

### Prioritization of conserved NPM1_Group1 regulators based on mouse AML models

**Fig. 5:**
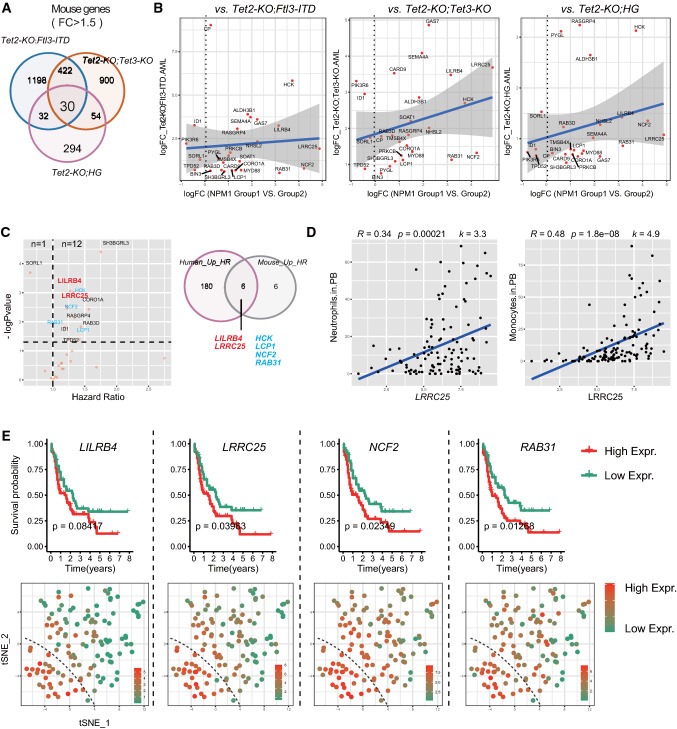
Transcriptomic datasets from murine AML models further assistants the screening of essential DEGs for characterizing NPM1_Group1 patients. **A** Venn diagrams of DEGs in three *TET2* mutation-related AML mouse datasets: *Tet2*^*−/−*^*; Tet3*^*−/−*^ (*Tet2*-KO; *Tet3*-KO)^*−*^, *Tet2*^*−/−*^*; Flt3ITD* (*Tet2*-KO; *Flt3ITD*) and *Tet2*^*−/−*^*; Ins2*^*Akita/*+^(*Tet2*-KO; HG). In total, 30 genes co-upregulated in the three murine AML models are prioritized (fold change > 1.5, p < 0.05). **B** Correlation plots of gene expression changes of transcriptomes from *NPM1*_Group1 vs. Group2 comparison of NIH-TCGA datasets and from murine sources of AML datasets. Each red dot represents a homologous gene. X-axis, logFC value from *NPM1*_Group1 vs Group2 comparison in the NIH-TCGA dataset; Y-axis, logFC value from the comparisons in murine datasets respectively. The blue line is a linear regression fitting based on all genes’ logFC value. Gray area: 95% confidence interval. **C** Among the 30 co-upregulated murine genes, the expression levels of 12 genes are positively correlated to AML patient survival while only 1 is negatively correlated. Finally, among the 12 HR-related murine genes, 6 are included in the 186-gene pool from Fig. 4C. **D** As a representative validation, *LRRC25* expression level is positively correlated with neutrophil and monocytes counts in PB of AML patients in Beat_AML datasets. **E** Validation of the 4 up-regulated risk genes in Kaplan–Meier survival plots and their expression profile in the *t*-SNE display of the entire AML pool. Dashed lines demarcate the boundary of FAB_M5 and FAB_M2 as same as in Fig. 2

**Fig. 6:**
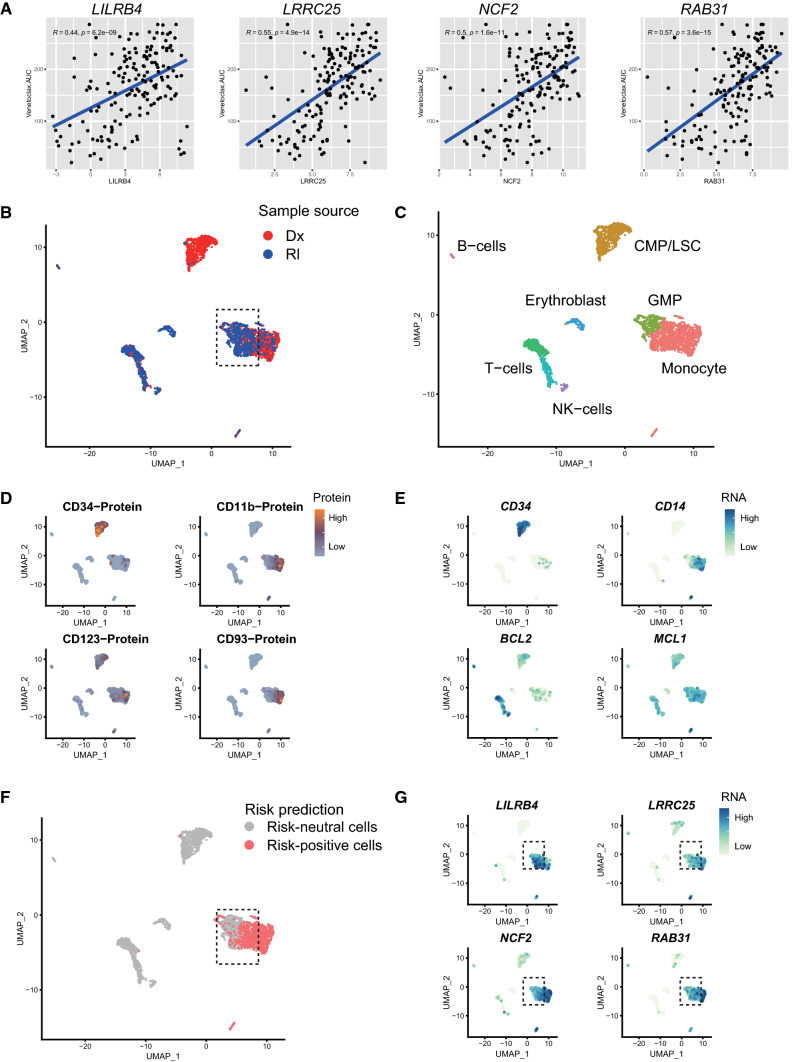
The four prioritized genes are potentially correlated with Venetoclax drug resistance in monocytic AML treatment. **A** The expression levels of the 4 prioritized genes are positively correlated with Venetoclax insensitivity indicated as area under curve (AUC) in the Beat_AML dataset. **B**–**G** At single cell level, the expression levels of the four prioritized genes are positively correlated with Venetoclax drug resistance in a longitudinal dataset of a monocytic AML patient. **B** BM cells of the patient are analyzed by cell surface labeling protein markers and RNA-seq at stages of both Diagnosis (Dx) and Relapse (Rl). **C** Although leukemic progenitors (CMP/LSC) were depleted by Venetoclax at the timepoint of Rl, other myeloid cells (GMP and monocytes) were persisted and the 4 prioritized gene were robustly expressed (see below). **D** and **E** Plots of representative markers in the Dx/Rl plots, including cell surface markers (i.e., CD34 and CD14) and apoptosis-related markers (i.e., BCL2 and MCL1). **F** Risk-positive cells were calculated in silico by Scissor algorithm (see Methods) and labeled as red dots (risk-neutral cells as gray dots). As shown in the plot, the risk-positive cells are mainly distributed in the GMP/monocytic pool rather than in the CMP/LSC pool. **G** At the single cell level, the risk-positive leukemic cells wildly and robustly express the four prioritized genes identified in Fig. 5. *CMP/LSC* common myeloid progenitor/leukemic stem cell, *GMP* granulocyte and megakaryocyte progenitor

Murine AML models provide another important resource for understanding AML disease onset and progression in a much more rigorous experimental setting. However, to what extent the murine models recapture human AML diseases and shared essential genes are largely unknown. We and other previously presented numerous animal models for *TET2*-related leukemia [12–14, 19, 20]. Transcriptome datasets of hematopoietic stem and progenitor cells from three AML mouse models *Tet2*^*−/−*^*;Flt3-ITD*, *Tet2*^*−/−*^*;Tet3*^*−/−*^ and *Tet2*^*−/−*^*;Ins2*^*Akita/*+^ were utilized here for further prioritization of essential genes. As shown in Fig. 5A, 30 co-upregulated DEGs were identified through overlapping analysis. Of note, the DEGs were based on the comparison between the leukemic models vs. wild type control respectively. Furthermore, most of the 30 DEGs were also upregulated in the *NPM1*_Group1 vs. Group2 (Fig. 5B). Their HR values were calculated and 12 genes have HR values greater than 1 (Fig. 5C). Finally, as shown in Figs. 5C, 6 out of the 12 genes were prioritized as they are also among the 186 HR-related DEGs in Fig. 4C: *LILRB4, LRRC25, NCF2, RAB31, LCP1* and *HCK*. Of note, 2 of the 6 genes encode membrane proteins: *LILRB4* and *LRRC25*.

We then validated the correlation between the 6 genes and FAB_M5 or general AML through different datasets. First, we analyzed the expression pattern of the 6 genes in various hematopoietic developmental stages using both pooled sample and single cell in the form of RNA-seq and ATAC-seq [21, 22]. As *LCP1* broadly expressed in all hematopoietic cells and *HCK* has been reported in myeloid leukemia, these two genes were not included in the following analysis [23–26]. The final prioritized genes, *LILRB4*, *LRRC25*, *NCF2* and *RAB31*, all are specifically expressed in myeloid mature cells or myeloid progenitors of healthy donors (Figure S2A, B and C). In leukemic datasets, these fours were also enriched in myeloid leukemia (Figure S2D). Interestingly, *LILRB4* is also enriched in bulk RNA-seq sample of acute lymphoid leukemia (Figure S2D).

Furthermore, based on the Beat_AML clinical datasets, the expression level of *LRRC25* was positively correlated with the count of neutrophils and monocytes in the peripheral blood of AML patients (Fig. 5D). To validate the prognosis of the 4 genes in the entire AML datasets, Kaplan–Meier survival analysis along with their expression pattern in *t*-SNE plot of TCGA_LAML cohort was performed. As shown in Fig. 5E, the expression level of *LRRC25*, *NCF2* and *RAB31* stratify entire AML patient pool with p value less than 0.05 (for *LILRB4*, p = 0.08). These results validate those three out of four genes have important and significant prognosis value in the TCGA dataset.

### Potential association of the four prioritized genes in Venetoclax drug resistance

The drug Venetoclax, an inhibitor of BCL2, has shown profound therapeutic efficacy in refractory AML treatment and been approved by FDA recently [7]. However, the drug resistance in both animal experimental and clinical setting were gradually recognized [8, 9]. For example, it has been reported that M4/M5 subtype of AML patients exhibit resistance to Venetoclax [8]. To explore the association between the four genes and Venetoclax, drug tailing datasets of the Beat_ AML cohort were extracted [10]. As initial evidence shown in Fig. 6A, the expression levels of *LILRB4, LRRC25, NCF2* and *RAB31* all are positively correlated with Venetoclax insensitivity.

Based on a scRNA-seq dataset with two disease stages: diagnosis and relapse after Venetoclax, we then explore the potential correlation of the four genes with Venetoclax drug resistance during treatment in a published dataset generated from a monocytic AML patient [8]. As shown in Fig. 6B and C, the application of the Venetoclax indeed effectively depleted leukemic progenitors, which were marked by high expression of CD34 protein and its messenger RNA (Fig. 6D and E). However myeloid progenitors at a lower hierarchy level and committed monocytic cells were resistant to the drug (the committed monocytes were labeled by CD11b and CD93; Fig. 6D and E). Using an algorithm (SCISSOR) for calculating the risk score of each single cell [27], we also validated that monocytic leukemic cells rather than leukemic progenitors are profoundly deleterious for diseases progression, as assessed by CD34/CD11b labeling and the in silico inferring (Fig. 6F). Finally, the high expression of the four genes were confirmed in the “risky” cells as shown in Fig. 6G, suggesting that deleterious leukemic cells could be marked by one of the four prioritized genes at single-cell resolution.

### LILRB4 overexpression promotes proliferation and Venetoclax resistance of leukemic cells in vitro

**Fig. 7:**
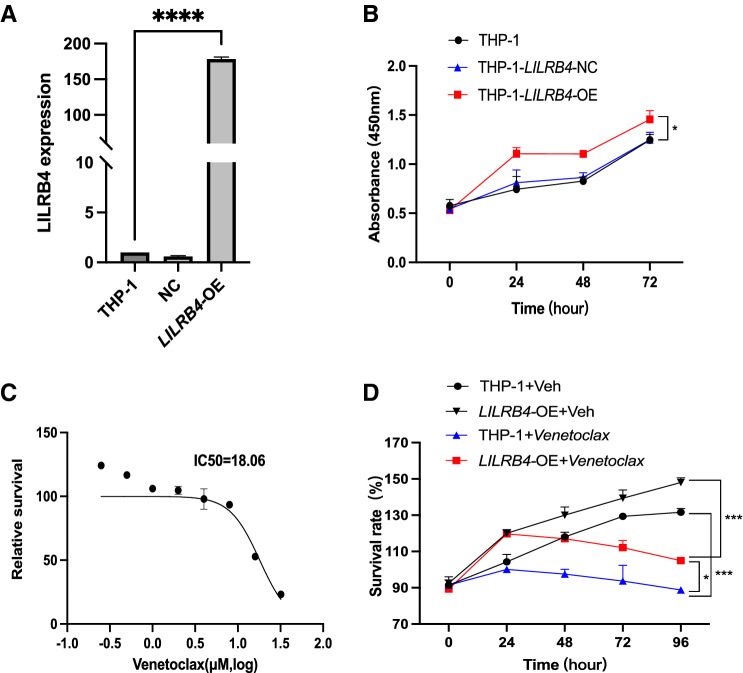
Overexpression of *LILRB4* in AML cell lines treated by Venetoclax. **A** Expression level of *LILRB4* in virus transfected THP-1 was determined by RT-PCR analysis. ****P < 0.0001. **B** Proliferation analysis of THP‑1 cells following transfection with *LILRB4* lentivirus vector, assessed by CCK-8. *P < 0.05; NC, negative control. **C** The IC-50 value of Venetoclax in THP-1 was determined by non-linear regression. **D** THP-1 cells with or without overexpression of *LILRB4* were treated with Venetoclax (18.06 μmol) over 96 h. The survival rates of each group were determined at 5 different time points. *P < 0.05

To verify the function of the four risk genes in AML and their resistance to Venetoclax, we chose *LILRB4* for in vitro validation since it was reported that LILRB4 is a therapeutic target of FAB M5 [28]. Expression levels of *LILRB4* were increased in the *LILRB4*-transfected THP-1 cells compared with the control (empty vector) (Fig. 7A). In a regular culture, *LILRB4* overexpression significantly promoted the proliferation of leukemia cells compared with the control (Fig. 7B). To assess if overexpression of *LILRB4* mediates the resistance to Venetoclax, we treated the THP-1 cells with Venetoclax. We determined the IC-50 value of Venetoclax is 18.06 μM (Fig. 7C**)** and then cultured the THP-1 cells in a medium with this concentration of Venetoclax. As shown in Fig. 7D, the cells with overexpression of *LILRB4* maintain increased survival rate compared to the control.

## Discussion

Overall, the present study is a comprehensive data-driven in silico research for prioritizing mammal-conserved risk genes (biomarkers), which may play essential role in diagnosis, progression and treatment management of AML, mainly in the form of FAB M5 subtypes. As outlined in Fig. 1, the datasets used in the study are divided into three major types of sources: (a) datasets from leukemia patients, TCGA_LAML for initial discovery while Beat_AML, KI_AML, Leucengene_AML and UHN_AML for validation; (b) datasets from mouse AML models (*Tet2-KO; Tet3-KO, Tet2-KO;Flt3-ITD, Tet2-KO; Ins2*^*Akita/*+^); and (c) datasets from healthy and other leukemic donors, including both pooled samples and single cell samples. The cross-center and cross-species usage of various datasets at the levels of both single patient or single cell warrant the outcome of our data-mining in a form with great conservation and accuracy. Since it has been recently reported that *LILRB4* is an immune therapeutic target for M5 AML [28, 29], the working flow and outcome will benefit further experimental and clinical validation of the other 3 novel risk genes (*LRRC25*, *NCF2* and *RAB31*), i.e. to access if they also function as therapeutic targets for monocytic AML as *LILRB4.*

Of note, there are no healthy donors in the TCGA_LAML cohort and our characterization had to mainly focus on comparing FAB_M5 vs. FAB_M2 or *NPM1*_Group1 vs. *NPM1*_Group2. Due to the challenging batch effect of transcriptomic data, we did not include GTEx datasets of healthy donors to the TCGA_LAML (data not shown). Concurrent collection of both leukemic samples and healthy samples are required in a future study design and it will further assist the prioritization of M2 or M5 features when compared with healthy donors.

In the dimension reduction for TCGA_LAML transcriptome datasets, we surprisingly found that *t*-SNE and UMAP clustering outperform the traditional PCA since they can discriminate M2, M3 and M5 in the 2D space (Fig. 2A). Similar outcomes were generated when analyzing datasets from other centers (Figure S1), confirming that the modern clustering methods are convenient and applicable in the future study once transcriptomics could be included for assisting diagnosis. Of note, the PCA algorithm is a traditional dimensional reduction that has been used for more than 100 years in mathematical analysis but *t*-SNE and UMAP dimension reduction are just recently developed and now wildly used in scRNA-seq data analysis with much better visualization intuition in 2D setting [17]. We also notice that for a hematopoietic system, *t*-SNE is as good as UMAP in many scenarios and it sometimes even can display relationship of biologically-related hematopoietic clusters as KNN algorithm for hematopoietic hierarchy [30]. As *t*-SNE and UMAP are well equipped in the Seurat and other single cell analysis software, cooperating these modern algorithms in bulk RNA-seq of patient samples will become very convenient and easy to be adopted for clinicians with limited high dimensional data-analyzing experience.

Since *t*-SNE and UMAP outperform well PCA in 2D visualization, a natural following question is why the modern dimension reduction methods discriminate so well for M2, M3 and M5? The answer probably underlies the distinct nature of the three subtypes of AML diseases which have different hematopoietic hierarchal programs, compared to that in healthy donors. This point of view is consistent with recent reports at a resolution of single-cell covering various subtypes of AML [30, 31]. Generally, FAB_M2 AML is with abundance of HSPC and lack of mature monocytes (primitive monocytes). FAB_M3 AML [32] is with abundance of promyeloblasts, which is at the development stage between HSPC and mature monocytes. In contrast, FAB_M5 AML typically has abundance of leukemic mature monocytes (committed monocytes). Stuck at different developmental stages indicate a distinct transcriptional program and transcriptome in the bulk RNA-seq samples. This may explain why *t*-SNE intuitively detects distinct expression pattern using high-dimension and demarcates the territory of FAB_M2, M3, M5 even although we are using pooled BM or PB cells. This explanation may also apply to why *t*-SNE cannot tell M4 from M5 and tell M2 from M0 or M1 in the 2D space because the M4 and M5 is quite similar in transcriptomic program and difference of M4 and M5 is not distinct enough in 2D setting. Discriminating M5 from M4 may have to rely on other inputs in clinic, i.e., stratification using single cell qualification rather than pooled PB or BM samples. It would be interesting to test if *t*-SNE plots reveal any stratifications in datasets documented with Europe Leukemia Network (ELN) diagnosis system.

Furthermore, as shown in Fig. 2B, stratification using transcriptomic data nevertheless outperforms the outcomes using mutation profiles. This is also understandable since transcriptomic data is profound, current, functional and comprehensive while genetic mutation profile is quiet and sometime indirect when another important parameter, variant allele fraction (VAF), is not considered at the same time. It has been recognized that VAF of several AML-related genes are dynamically changed in the set of clonal hematopoiesis in healthy donors or leukemic patients [33]. Greater VAF certainly indicates greater tumor mutation burden otherwise the impact of mutations will be minimized and even not be reflected at the transcriptomic space. In conclusion, the present study highlights that using transcriptomic data can largely assist the precisive diagnosis and stratification of highly heterogeneous diseases, especially when the transcriptomic information was integrated with mutation profiles and clinical observations. Considering that today the cost of obtaining transcriptomic information (bulk RNA-seq of peripheral blood) is very low, the input of such information will greatly assist diagnosis.

After several rounds of filtering and prioritization, six genes were finally presented with conservation and positive HR value: *LILRB4, LRRC25, NCF2, RAB31, LCP1 and HCK.* Interestingly five of the gene are specifically expressed in myeloid cells or progenitors, except that *LCP1* is expressed in both myeloid lineage and lymphoid lineage (Fig. 6 and S2, and data not shown). Furthermore, published reports suggest that all the encoded proteins are involved in signaling-related in immune response, indicating they may have important role in cancer immunity or innate immunity. Indeed, it has been recently reported that *LILRB4* is a novel immune therapy target for FAB_M5 AML [28]. The role of *LCP1* and *HCK* in leukemia or lymphoma has been sporadically reported [23–26]. However, role of *LRRC25, NCF2* and *RAB31* is largely unknown and the experimental validation are demanded in future study to facilitate these proteins used in clinic [34–38]. As an initial verification, we have validated that the high expression of four genes is associated with Venetoclax drug resistance at pooled cell level of PB in Beat_AML corhot and also at single cell level in the longitudinal relapsed BM sample of a M5 patient (Fig. 6). More single cell-style datasets of FAB_M5 patient and experimental verification in vitro and in vivo are required for further characterizing the role of the protein in Venetoclax drug resistance.

In summary, the present in silico study integrated several aspects of AML datasets, including transcriptome, mutation, clinical information, single cell data, risk stratification with overall survival. We finally prioritize and validate four immune signaling-related genes as novel markers for FAB_M5 AML. The working flow and outcome will also guide future research and stratification analysis of other diseases such as autoimmune disorders and other types of blood cancers.

## Supplementary Information

**Additional file 1.**

**Additional file 2.**

**Additional file 3.**

**Additional file 4.**

## Data Availability

The dataset including different cohorts of AML patients and three different AML mice model data. Human datasets, murine datasets, drug AUC dataset, scRNA-seq datasets and scATAC-seq dataset are shown in Supplemental Table 1.
