# Correction to: Prioritizing risk genes as novel stratification biomarkers for acute monocytic leukemia by integrative analysis

**DOI:** 10.1007/s12672-022-00526-w

**Published:** 2022-09-26

**Authors:** Hang He, Zhiqin Wang, Hanzhi Yu, Guorong Zhang, Yuchen Wen, Zhigang Cai

**Affiliations:** 1 Department of Hematology, Tianjin Medical University General Hospital, Tianjin, China; 2 Department of Rheumatology, Tianjin Medical University General Hospital, Tianjin, China; 3 The Province and Ministry Co-Sponsored Collaborative Innovation Center for Medical Epigenetics, Department of Pharmacology, School of Basic Medical Science, Tianjin Medical University, Tianjin, China

## Correction to: Discover Oncology 13:55 (2022) https://doi.org/10.1007/s12672-022-00516-y

The original publication of this article [1] has been updated to correct 2 problems:

1. The affiliation list has been reordered
2. The text “A and data not shown” in the caption of Fig. 1 has been updated to “Fig. 2A and data not shown”
